# An analysis of protective health behavior and polypharmacy among older patients: a nationwide cohort study

**DOI:** 10.1186/s12877-024-05207-7

**Published:** 2024-07-30

**Authors:** Zhaoyan Piao, Kyung sun Oh, Euna Han

**Affiliations:** 1 College of Pharmacy, Yonsei Institute of Pharmaceutical Sciences, Yonsei University, 162-1 Songdo-Dong, Yeonsu-Gu, Incheon, Republic of Korea; 2 College of Pharmacy, Dongduk Women’s University, Hwarang-ro 13, Seongbuk-gu, Seoul, Republic of Korea

**Keywords:** Polypharmacy, Protective health behavior, Cohort study, BMI, Smoking

## Abstract

**Background:**

This study analyzed the relationship between protective health behaviors and polypharmacy in individuals aged 65 years and older.

**Methods:**

We used data from a nationwide survey (KNHANES) from 2012 to 2016 in conjunction with the health insurance claims databases. A total of 3297 adults aged 65 or older were included in the study. Polypharmacy was defined as more than 30 prescription days in 6 months with five or more different drugs. Health-related behaviors (BMI, smoking, drinking, regular walking, and living alone) were extracted for 6 months before measuring polypharmacy. We used multivariable logistic regression on polypharmacy for each protective health behavior, as well as a composite score of protective health behavior. Subgroup analysis was also conducted by age and sex.

**Results:**

Among protective health behaviors, BMI < 25 (OR, 0.76; 95% CI, 0.66–0.88) and never smoking (OR, 0.78; 95% CI, 0.62–0.98) were associated with a lower risk of polypharmacy. Polypharmacy was significantly associated with BMI < 25 in both sex subgroups (male: OR, 0.71; 95% CI, 0.56–0.88; female: OR, 0.81; 95% CI, 0.67–0.99) and 65–79 subgroup (OR, 0.74; 95% CI, 0.63–0.86). The association between never smoking and polypharmacy was only significant in the 65–79 subgroup (OR, 0.71; 95% CI, 0.55–0.91). Participants with five protective health behaviors had a lower risk of polypharmacy than participants with zero or one health behavior, which was only statistically significant in the subgroup analysis of participants aged 65–79 years (OR, 0.52; 95% CI, 0.29–0.94).

**Conclusions:**

This study finds that health behaviors such as obesity and smoking are associated with a higher risk of polypharmacy. Furthermore, we confirm that a high score of protective health behaviors is associated with a lower risk of polypharmacy. Our findings indicate the need for geriatric-centered management of protective health behaviors to prevent polypharmacy.

**Supplementary Information:**

The online version contains supplementary material available at 10.1186/s12877-024-05207-7.

## Background

The older adult population in South Korea has been rapidly increasing, reaching 11.9% in 2013, and is projected to escalate to 25.3% by 2030, thereby transitioning the country into an ultra-aged society [1]. Older adults exhibit a higher prevalence of chronic diseases due to a physiological decline of various organ functions compared to other age groups [2]. Therefore, concurrent use of multiple medications becomes essential for older patients with two or more chronic conditions, and polypharmacy is more prevalent among older populations with multimorbidity [3]. 

Polypharmacy refers to the prescription of five or more medications [4, 5]. This represents inappropriate and excessive prescription practices [6, 7]. Previous research using the National Health and Nutrition Examination Survey in the United States revealed that 45.1% of individuals aged 65 and older received prescriptions for five or more medications [8]. Similarly, a study conducted in Japan in 2015 found that 28.7% of individuals aged 65 years and older were prescribed five or more medications [9]. A study based on claims data revealed that 44% of seniors aged 65 years or older in Korea were taking five or more prescribed medications [10]. 

Prescribing cascades frequently occur with polypharmacy, where the side effects of one medication may be misconstrued as symptoms of another condition. Consequently, prescribing additional medications to address these perceived symptoms may lead to the occurrence of further adverse effects [11, 12]. Polypharmacy is also associated with decreased medication adherence and drug–drug interactions, leading to social issues such as increased hospitalizations and medical costs [13–16]. 

Research investigating the causal link between protective health behaviors and polypharmacy in older adults is limited. Previous research on polypharmacy among older adults focused on investigating the association between inappropriate medication prescribing and adverse outcomes, such as falls, cognitive impairment, and mortality [16, 17]. A few studies have explored the prevalence of polypharmacy among older adults, although most of those studies only used insurance claims data, lacking socioeconomic data and protective health behaviors potentially associated with polypharmacy among older adults in Korea [18, 19]. 

This study analyzed the relationship between protective health behaviors and polypharmacy for individuals aged 65 years and older by integrating health behavior information from the nationally representative Korea National Health and Nutrition Examination Survey and medication insurance claims data from the National Health Insurance Service, compared to previous research solely reliant on health behavior data from the general population based on surveys [20]. Furthermore, we estimated risk factors associated with polypharmacy by integrating protective health behaviors with demographic characteristics. We considered health-related behaviors associated with polypharmacy, including physical activity, smoking, and alcohol consumption [21] alongside key risk indicators for chronic diseases such as body mass index (BMI) [22] in this study. Moreover, the study considered the rising trend of living alone in Korea [23]. This approach allowed for a comprehensive analysis of protective health behaviors and demographic profiles in predicting polypharmacy. The results allow us to predict the risks associated with polypharmacy across various older adult age groups, which makes analyzing appropriate management standards feasible.

## Methods

### Data source

We used data collected by the Korea National Health and Nutrition Examination Survey (KNHANES) [24] from 2012 to 2016 in conjunction with the National Health Insurance Service (NHIS) administrative claims database and Health Insurance Review and Assessment Service–National Patient Sample (HIRA-NPS) data. The KNHANES is a nationwide survey performed regularly by the Korea Centers for Disease Control and Prevention (KCDC) to explore the health status of the South Korean population through health and nutrition interviews and a basic health assessment [25]. Participants are selected by proportionate allocation-systematic sampling with multistage stratification using age, gender, residence area, education level, and other characteristics [25]. The NHIS is Korea’s sole health insurance provider, covering the entire Korean population. The NHIS dataset contains the beneficiaries’ qualification information, including disability status and death. Korean healthcare providers have submitted claims on medical services to HIRA for review and reimbursement since 2000. Accordingly, the HIRA database contains information on reimbursement for medical care used by the Korean population.

Ethical approval and an informed consent waiver to manage retrospective data were approved by the Institutional Review Board of Inha University Hospital (2022-09-039-001).

### Study population

**Fig. 1:**
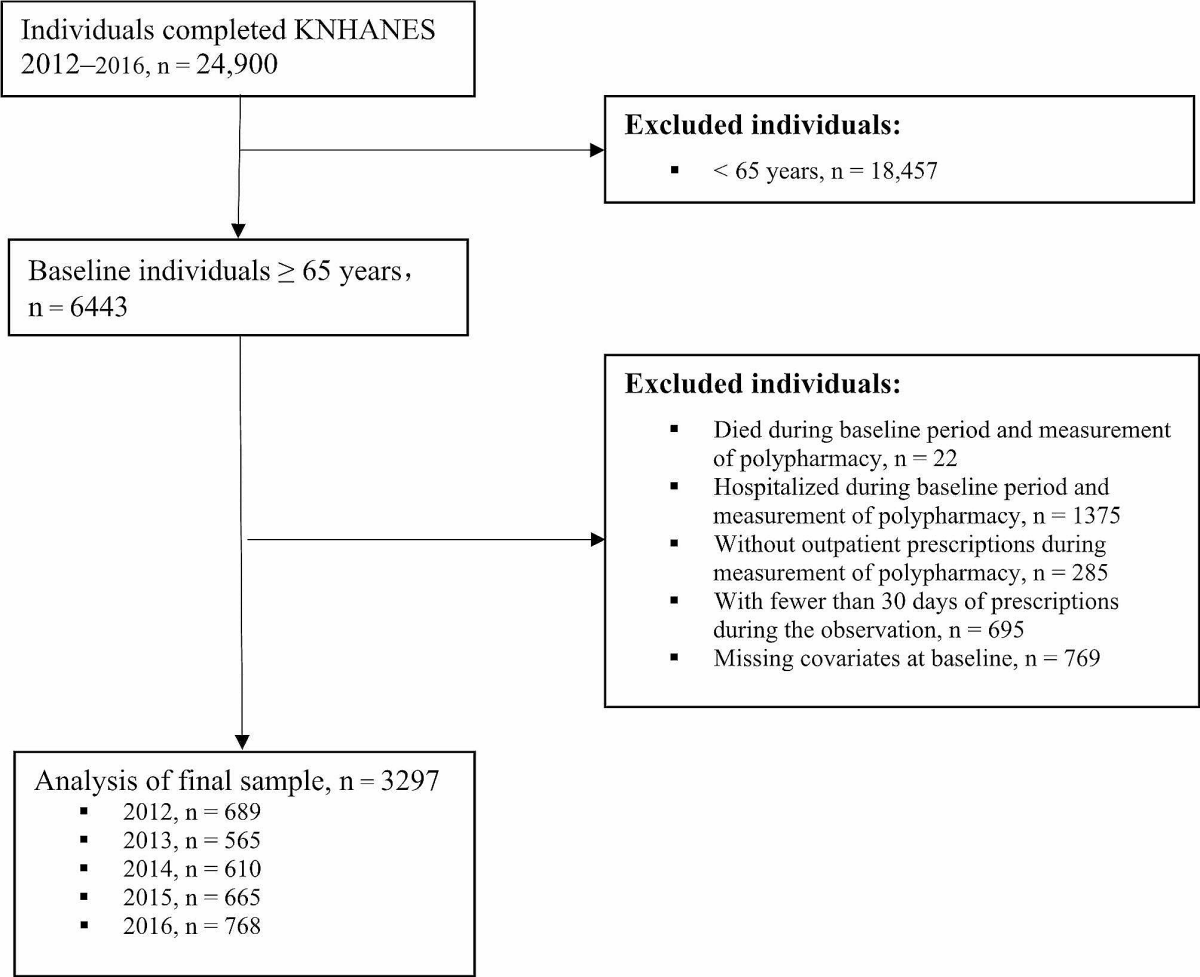
Flow diagram of the sample selection process

There were 24,900 participants in the KNHANES during 2012–2016, and we excluded 18,457 individuals aged under 65 years. Additionally, 1397 people were excluded from the study because they died or were hospitalized during the baseline period (6 months before the index date since polypharmacy was measured) or the period for polypharmacy measurement. Another 980 people were excluded because they did not have any outpatient prescriptions or had fewer than 30 days of prescriptions during the measurement of polypharmacy. Furthermore, 769 individuals were excluded due to missing variables. Finally, 3297 older adults were included in our study (Fig. 1).

### Definition and measurement

#### Polypharmacy

In this study, polypharmacy was defined from claims data as more than 30 prescription days with five or more different drugs in six months (Appendix Fig. 1) [4, 26, 27]. We recorded the Korean national drug code according to the WHO-Anatomical Therapeutic Chemical (ATC) Classification System to measure the number of drugs [28]. 

#### Protective health behaviors

The relevant variables regarding protective health behaviors were extracted from the KNHANES questionnaire during the 6 months before the index date because polypharmacy was measured over 6 months. We defined five protective health behaviors: never smoking, consuming fewer than 7 drinks twice a week, [29] walking more than or equal to 5 days per week, not living alone, and body mass index (BMI; kg/m^2^) < 25. In addition, we generated a new variable based on the number of protective health behaviors engaged in [21, 30]. 

#### Other covariates

Other covariates included sex, age, Charlson comorbidity index (CCI) weighted to include 12 chronic conditions [31–33], residence area, household income quartile, health insurance type, private medical insurance, having undergone a health screening within the previous 2 years, having undergone a cancer screening within the last 2 years, education level, usual perception of stress, calendar year, and disability. The data on disability were obtained from claims data, and data on the remaining variables were obtained from KNHANES.

Age was separated into two groups: 65–79 and ≥ 80 years old. CCI was identified by extracting primary diagnoses from claims data according to ICD-10 codes. Based on the 12 chronic conditions, they were categorized into three groups: 0, 1, and 2 or more. Residence areas were classified into three groups based on population density: metropolitan, urban, and rural. The income quartiles (households) were low, lower-middle, upper-middle, and high. Education level was divided into two categories: less than high school and high school and above. Health insurance was divided into two types: National Health Insurance and Medical Aid. The calendar year denotes the year the survey was completed.

### Statistical analyses

We used frequency analysis and chi-square tests to explore the associations between demographic characteristics and polypharmacy. Then, we analyzed the proportion of polypharmacy according to the number of protective health behaviors and performed subgroup analysis by age and sex. Finally, we estimated multivariable regressions controlling for the aforementioned covariates. In Model 1, we used multivariable logistic regression to estimate odds ratios (ORs) for polypharmacy and their 95% confidence intervals (CIs) to estimate the odds ratio for each protective health behavior as predictive factors for polypharmacy. In Model 2, we estimated the relative odds of the number of protective health behaviors associated with polypharmacy.

All statistical analyses were performed using SAS 9.4. A two-sided test of *P* < 0.05 was considered statistically significant.

## Results

A total of 3297 individuals were included in the analysis, comprising 1437 individuals without polypharmacy and 1860 individuals with polypharmacy. Age distribution varied significantly (*P* < 0.05), with individuals aged 65–79 years accounting for a higher proportion of the polypharmacy group (85.27%) than the non-polypharmacy group (88.73%). Similarly, significant differences were found in sex distribution (*P* < 0.05), with females accounting for a higher proportion of the polypharmacy group than the no polypharmacy group (57.96% vs. 50.87%). A greater proportion of individuals with polypharmacy were low-income than those without polypharmacy (49.41% vs. 44.61%). More individuals without polypharmacy presented a CCI score of 0 (by 15.05% points) but a lower disability rate (by 5.65% points) than the no polypharmacy group. People with polypharmacy were more likely to live alone (22.96% vs. 19.49%), have a BMI ≥ 25 (64.72% vs. 58.39%), walk < 5 days a week (41.61% vs. 45.37%), and drink excessively (3.13% vs. 2.96%), and were less likely never to have smoked (39.73% vs. 42.10%) (Table 1).

**Table 1:** Characteristics of study population

Variables	All (*n* = 3297)		No polypharmacy (*n* = 1437)		Polypharmacy (*n* = 1860)		*P*-value
	*N*	%	*N*	%	*N*	%	
**Age (years)**							< 0.05
65–79	2861	86.78	1275	88.73	1586	85.27	
≥ 80	436	13.22	162	11.27	274	14.73	
**Sex**							< 0.05
Male	1488	45.13	706	49.13	782	42.04	
Female	1809	54.87	731	50.87	1078	57.96	
**Living alone**							< 0.05
Yes	707	21.44	280	19.49	427	22.96	
No	2590	78.56	1157	80.51	1433	77.04	
**BMI**							< 0.05
< 25	2016	61.15	930	64.72	1086	58.39	
≥ 25	1281	38.85	507	35.28	774	41.61	
**Walking days per week**							< 0.05
< 5	1871	56.75	785	54.63	1086	58.39	
≥ 5	1426	43.25	652	45.37	774	41.61	
**Ever smoked**							0.17
Yes	1953	59.24	832	57.90	1121	60.27	
No	1344	40.76	605	42.10	739	39.73	
**Drink**							0.77
Excessive	100	3.03	45	3.13	55	2.96	
Non-excessive	3197	96.97	1392	96.87	1805	97.04	
**Residential area**							0.12
Metropolitan	1501	45.53	669	46.56	832	44.73	
Urban	1033	31.33	460	32.01	573	30.81	
Rural	763	23.14	308	21.43	455	24.46	
**Income quartile (household)**							< 0.05
Low	1560	47.32	641	44.61	919	49.41	
Lower-middle	929	28.18	419	29.16	510	27.42	
Upper-middle	474	14.38	208	14.47	266	14.30	
High	334	10.13	169	11.76	165	8.87	
**Health insurance type**							< 0.05
National health insurance	3090	93.72	1385	96.38	1705	91.67	
Medical aid	207	6.28	52	3.62	155	8.33	
**Private medical insurance**							< 0.05
No	1137	34.49	554	38.55	583	31.34	
Yes	2160	65.51	883	61.45	1277	68.66	
**Health screening within 2 years**							0.78
No	2233	67.73	977	67.99	1256	67.53	
Yes	1064	32.27	460	32.01	604	32.47	
**Cancer screening within 2 years**							0.93
No	2083	63.18	909	63.26	1174	63.12	
Yes	1214	36.82	528	36.74	686	36.88	
**Education level**							< 0.05
Less than high school	2429	73.67	1002	69.73	1427	76.72	
High school and above	868	26.33	435	30.27	433	23.28	
**Charlson comorbidity index**							< 0.05
0	2205	66.88	1083	75.37	1122	60.32	
1	531	16.11	190	13.22	341	18.33	
≥ 2	561	17.02	164	11.41	397	21.34	
**Disability**							< 0.05
No	2818	85.47	1274	88.66	1544	83.01	
Yes	479	14.53	163	11.34	316	16.99	
**Usual perception of stress**							< 0.05
No	2671	81.01	1197	83.30	1474	79.25	
Yes	626	18.99	240	16.70	386	20.75	
**Calendar year**							0.84
2012	689	20.90	313	21.78	376	20.22	
2013	565	17.14	241	16.77	324	17.42	
2014	610	18.50	267	18.58	343	18.44	
2015	665	20.17	288	20.04	377	20.27	
2016	768	23.29	328	22.83	440	23.66	

**Fig. 2:**
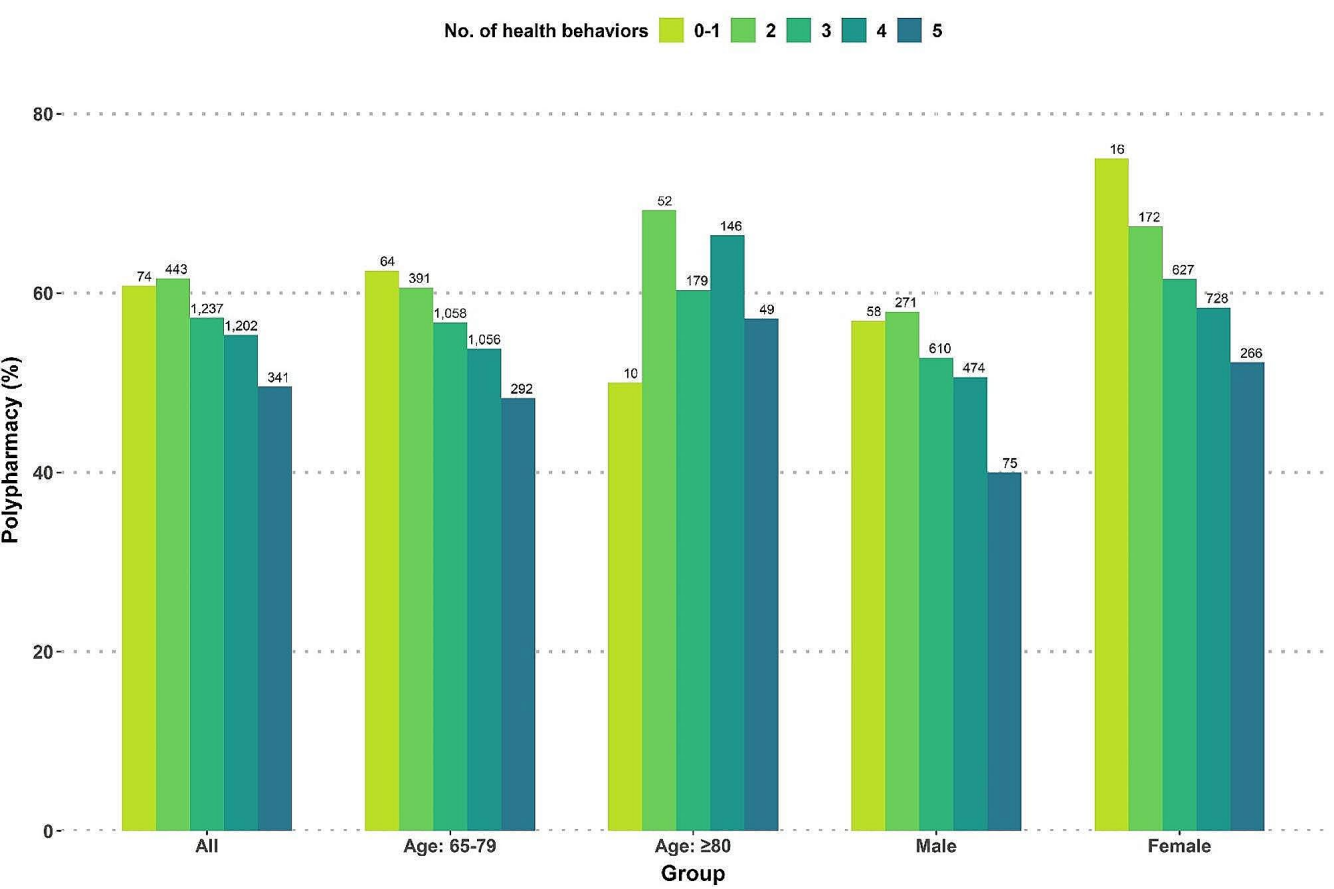
Percentages of polypharmacy based on the number of protective health behaviors

Figure 2 shows the prevalence of polypharmacy according to the number of protective health behaviors. The composite score of protective health behaviors and the polypharmacy were inversely related across gender and age subgroups except for the 80 years and older subgroup.

The number of people with 3 (*n* = 1237) or 4 (*n* = 1202) categorized protective health behaviors was the highest, and the number of people with 0–1 protective health behaviors was the lowest (*n* = 74). As the number of protective health behaviors increased, the prevalence of polypharmacy decreased from 60.81% for 0–1 to 49.56% for five protective health behaviors. A similar trend was observed in the 65–79 age group. However, the trend differed among those aged 80 years or older: polypharmacy prevalence was only 50% for those with 0–1 protective health behaviors, while, for 2 + health behaviors, it was higher than among those aged 65–79 years. The gender subgroup analysis showed that the prevalence of polypharmacy was higher among women than men, regardless of the number of protective health behaviors.

Table 2 shows the relationships between polypharmacy and protective health behavior clusters. In Model 1, those with a BMI < 25 had lower odds of polypharmacy compared with those with a BMI ≥ 25 (OR, 0.76; 95% CI, 0.66–0.88). This difference remained significant among those aged 65–79 years (OR, 0.74; 95% CI, 0.63–0.86) but not those aged 80 years or older (OR, 0.92; 95% CI, 0.57–1.47). Moreover, the sex subgroup analysis also showed a significant difference, especially among men (male: OR, 0.71; 95% CI, 0.56–0.88; female: OR, 0.81; 95% CI, 0.67–0.99). Never having smoked was negatively associated with polypharmacy (OR, 0.78; 95% CI, 0.62–0.98), but the association was only significant in those aged 65–79 years (OR, 0.71; 95% CI, 0.55–0.91). In addition, not living alone (OR, 1.02; 95% CI, 0.84–1.23), not drinking excessively (OR, 0.95; 95% CI, 0.63–1.44), and walking more than or equal to 5 days a week (OR, 0.94; 95% CI, 0.81–1.08) were not significantly associated with polypharmacy, including in the subgroup analyses of age and sex.

**Table 2:** Association between behavioral variables and polypharmacy with multivariable logistic regression

	Dependent variable: polypharmacy										
	**All**		**65–79 years**		**≥ 80 years**			**Male**		**Female**	
	**OR**	**95% CI**	**OR**	**95% CI**	**OR**	**95% CI**	**OR**		**95% CI**	**OR**	**95% CI**
Model 1											
Not living alone	1.02	0.84–1.23	0.99	0.80–1.21	1.13	0.68–1.89	1.26		0.87–1.82	0.92	0.74–1.15
BMI < 25	**0.76**	**0.66–0.88**	**0.74**	**0.63–0.86**	0.92	0.57–1.47	**0.71**		**0.56–0.88**	**0.81**	**0.67–0.99**
Non-excessive drinker	0.95	0.63–1.44	0.90	0.59–1.37	-	-	0.97		0.63–1.48	0.59	0.06–5.81
Never smoked	**0.78**	**0.62–0.98**	**0.71**	**0.55–0.91**	1.17	0.66–2.06	0.79		0.60–1.03	0.75	0.50–1.14
Walk ≥ 5 days a week	0.94	0.81–1.08	0.96	0.83–1.12	0.77	0.50–1.18	0.97		0.78–1.20	0.90	0.74–1.10
Age > 80	1.30	1.04–1.63	-	-	-	-	1.58		1.10–2.28	1.13	0.84–1.52
Female	1.52	1.19–1.92	1.68	1.29–2.18	0.98	0.51–1.82	-		-	-	-
Urban	1.00	0.85–1.18	0.89	0.75–1.06	2.20	1.33–3.63	1.03		0.81–1.32	0.97	0.78–1.22
Rural	1.14	0.95–1.37	1.12	0.92–1.38	1.30	0.77–2.20	1.15		0.87–1.52	1.11	0.86–1.42
Income quartile (household): Lower-middle	1.01	0.85–1.21	1.03	0.85–1.25	0.99	0.58–1.77	0.97		0.75–1.26	1.07	0.84–1.38
Income quartile (household): Upper-middle	1.15	0.91–1.44	1.14	0.90–1.48	1.22	0.58–2.54	1.05		0.75–1.46	1.30	0.94–1.79
Income quartile (household): High	0.94	0.72–1.23	0.94	0.71–1.24	1.12	0.39–3.28	0.93		0.64–1.35	0.97	0.66–1.43
Charlson comorbidity index = 1	1.88	1.38–2.06	1.62	1.30–2.01	1.98	1.14–3.45	1.92		1.43–2.57	1.49	1.13–1.96
Charlson comorbidity index ≥ 2	2.38	1.94–2.92	2.26	1.82–2.80	4.35	2.08–9.20	2.66		1.99–3.55	2.15	1.61–2.87
Medical aid	1.89	1.34–2.66	1.90	1.30–2.78	1.68	0.72–3.91	1.67		1.02–3.06	2.02	1.32–3.08
Without private medical insurance	0.81	0.69–0.95	0.81	0.68–0.95	0.87	0.28–1.62	0.84		0.65–1.07	0.78	0.63–0.97
Health screening within 2 years	1.05	0.81–1.35	1.09	0.82–1.45	0.75	0.40–1.41	1.06		0.73–1.47	1.03	0.71–1.47
Cancer screening within 2 years	1.09	0.85–1.40	1.05	0.79–1.38	1.41	0.74–2.69	1.01		0.71–1.53	1.17	0.81–1.87
High school education and above	0.85	0.71-1.00	0.87	0.72–1.06	0.75	0.41–1.39	0.92		0.73–1.13	0.74	0.55–0.99
Disability	1.48	1.20–1.83	1.64	1.30–2.06	0.93	0.52–1.67	1.44		1.08–1.93	1.58	1.15–2.16
Usual perception of stress	1.16	0.98–1.40	1.28	1.04–1.58	0.57	0.33–0.98	1.06		0.76–1.47	1.20	0.95–1.51
Calendar year: 2013	1.11	0.88–1.40	1.13	0.88–1.45	0.95	0.47–1.91	1.07		0.75–1.52	1.15	0.84–1.57
Calendar year: 2014	1.12	0.89–1.43	1.07	0.84–1.36	1.71	0.85–3.46	1.21		0.86–1.71	1.05	0.78–1.42
Calendar year: 2015	1.15	0.92–1.44	1.14	0.90–1.45	1.09	0.56–2.12	1.22		0.88–1.71	1.10	0.81–1.48
Calendar year: 2016	1.16	0.93–1.44	1.22	0.97–1.53	0.88	0.47–1.85	1.31		0.94–1.82	1.07	0.80–1.43
Model 2											
No. of protective health behaviors											
0–1	Ref.		Ref.		Ref.		Ref.			Ref.	
2	1.04	0.62–1.73	0.90	0.52–1.57	2.49	0.59–10.59	1.11		0.62-2.00	0.82	0.25–2.72
3	0.86	0.53–1.41	0.77	0.45–1.32	1.48	0.39–5.71	0.93		0.53–1.61	0.67	0.21–2.17
4	0.79	0.48–1.30	0.67	0.39–1.15	1.94	0.49–7.61	0.85		0.49–1.49	0.61	0.19–1.98
5	0.61	0.36–1.05	**0.52**	**0.29–0.94**	1.38	0.32–5.94	0.57		0.28–1.16	0.50	0.15–1.64
Age > 80	1.27	1.02–1.59	**-**	**-**	-	-	1.54		1.07–2.17	1.12	0.84–1.50
Female	1.38	1.18–1.62	1.46	1.23–1.73	1.02	0.63–1.66	-		-	-	-
Urban	0.99	0.84–1.17	0.89	0.74–1.06	2.23	1.35–3.66	1.03		0.80–1.31	0.96	0.77–1.21
Rural	1.12	0.93–1.35	1.10	0.90–1.35	1.42	0.83–2.43	1.15		0.87–1.52	1.09	0.85–1.41
Income quartile (household): Lower-middle	1.04	0.87–1.24	1.05	0.87–1.27	1.12	0.64–1.93	1.00		0.77–1.30	1.08	0.85–1.37
Income quartile (household): Upper-middle	1.19	0.95–1.49	1.19	0.94–1.51	1.32	0.65–2.66	1.10		0.79–1.52	1.32	0.97–1.81
Income quartile (household): High	0.98	0.75–1.28	0.97	0.74–1.28	1.20	0.42–3.46	0.96		0.66–1.40	1.00	0.68–1.46
Charlson comorbidity index = 1	1.69	1.38–2.06	1.63	1.31–2.02	2.01	1.16–3.50	1.92		1.43–2.57	1.50	1.14–1.98
Charlson comorbidity index ≥ 2	2.38	1.94–2.92	2.27	1.83–2.81	4.37	2.07–9.20	2.68		2.01–3.58	2.15	1.61–2.87
Medical aid	1.84	1.31–2.58	1.86	1.27–2.71	1.69	0.73–3.96	1.49		0.83–2.68	2.02	1.33–3.07
Without private medical insurance	0.81	0.69–0.95	0.81	0.69–0.95	0.70	0.29–1.68	0.84		0.66–1.06	0.78	0.63–0.98
Health screening within 2 years	1.05	0.81–1.35	1.10	0.83–1.46	0.75	0.40–1.42	1.03		0.73–1.54	1.02	0.71–1.47
Cancer screening within 2 years	1.10	0.85–1.41	1.05	0.80–1.38	1.38	0.72–2.63	1.02		0.72–1.45	1.17	0.81–1.68
High school education and above	0.85	0.71–1.02	0.87	0.72–1.08	0.73	0.40–1.34	0.93		0.74–1.17	0.73	0.54–0.99
Disability	1.48	1.20–1.83	1.63	1.29–2.04	0.96	0.54–1.72	1.42		1.07–1.90	1.58	1.16–2.16
Usual perception of stress	1.16	0.96–1.39	1.27	1.04–1.55	0.55	0.32–0.95	1.03		0.74–1.43	1.20	0.96–1.51
Calendar year: 2013	1.11	0.88–1.40	1.12	0.88–1.44	0.95	0.47–1.93	1.06		0.74–1.51	1.15	0.84–1.58
Calendar year: 2014	1.12	0.90–1.41	1.08	0.85–1.37	1.72	0.85–3.45	1.21		0.86–1.70	1.08	0.78–1.43
Calendar year: 2015	1.15	0.92–1.43	1.15	0.90–1.47	1.08	0.55–2.11	1.20		0.86–1.67	1.10	0.81–1.48
Calendar year: 2016	1.16	0.93–1.43	1.20	0.96–1.52	0.90	0.48–1.70	1.26		0.91–1.75	1.08	0.81–1.44

In Model 2, using people with 0–1 protective health behaviors as the reference, the odds of polypharmacy for people with 2, 3, 4, and 5 protective health behaviors were not significant (2 protective health behaviors: OR, 1.04; 95% CI, 0.62–1.73; 3 protective health behaviors: OR, 0.86; 95% CI, 0.53–1.41; 4 protective health behaviors: OR, 0.79; 95% CI, 0.48–1.30; 5 protective health behaviors: OR, 0.61; 95% CI, 0.36–1.05). In the 65–79 years subgroup, the aggregation of five protective health behaviors (versus 0–1) was associated with a lower risk of polypharmacy (OR, 0.52; 95% CI, 0.29–0.94). Additionally, the odds of polypharmacy were higher among females, individuals aged 80 years and older, people on Medical Aid, those without private health insurance, and those with a lower level of education (Table 2).

## Discussion

This study assessed the relationship between health-related behaviors and polypharmacy in individuals aged 65 years and older. Given that polypharmacy is more prevalent among older populations with multiple morbidities, [3] prescribing cascades may lead to various adverse effects [11, 12] and healthcare service use [13–16]. Our study revealed that older adults who engaged in protective health behaviors and those living with two or more family members tend to have a lower rate of polypharmacy. Specifically, for older adults aged 65–79, having a BMI under 25 and never smoking showed statistically significant lower odds of polypharmacy. When evaluating the risk of polypharmacy based on a composite score of protective health behaviors, the risk among older adults aged 65 to 79 who engaged in all five protective health behaviors was approximately 50% lower than that of older adults engaging in 0–1 behaviors.

Our results are consistent with previous studies that assessed health risk factors influencing healthcare utilization through National health screening data and National Health Insurance cohort data [30]. Previous studies targeting general populations also showed that health-related risk factors influencing healthcare utilization, such as obesity, smoking—alone or in combination with obesity—and obesity with alcohol consumption, were significant contributors to healthcare utilization, including admission visits and medical costs [30]. However, conflicting results in the literature necessitate interpreting the impact of obesity in older adult populations carefully. Previous studies in Korea utilizing NHIS data have shown a U-shaped relationship between mortality rates and obesity, with lower mortality rates among individuals in the overweight range (BMI 25-26.4) [34]. Because BMI cannot accurately distinguish between fat and muscle mass, overweight based on BMI may be paradoxically associated with positive health outcomes, while obesity in older adults may be a risk factor for polypharmacy. Therefore, while obesity may serve as a risk factor for polypharmacy in older adults, it may also be associated with positive health outcomes.

This study’s findings align with previous research findings indicating that composite healthy behavior scores, such as physical activity, smoking abstinence, and avoidance of sedentary behavior, are inversely associated with all-cause mortality [35]. Additionally, a consistent trend observed in previous research suggests a relationship between health behaviors, including smoking cessation, physical activity, and adherence to a healthy diet, and the risk of polypharmacy and hospitalization [21]. Our results align with previous research [21, 35] in finding that healthy behaviors could be an important factor for predicting polypharmacy and related healthcare service use. In addition, our data included variables on alcohol use ever and exercise intensity. However, given that our study sample comprised of people aged 65 years and older, these variables were considered unsuitable because of few variations in those variables.

Conversely, our results indicate that the risk of polypharmacy among older adults who are Medical Aid recipients was higher compared to those covered by National Health Insurance. Previous research on individuals aged 30 and above in Korea indicates that polypharmacy is more prevalent not only among low-income groups who experience minimal economic burden for medical utilization due to the benefits of the healthcare system but also among high-income groups with sufficient payment ability for medical services [18]. The higher rate of polypharmacy among Medical Aid patients may be partially attributed to the lower burden of medical utilization expenses compared to National Medical Insurance patients. However, polypharmacy also appears to be associated with demographic characteristics that are more prevalent among Medical Aid beneficiaries, including older age and multiple comorbidities [36]. 

Our results demonstrate that older adults with two or more concurrent comorbidities representing complex medical conditions had a higher risk of polypharmacy. To address potential confounding factors related to multiple comorbidities, we investigated the changes in coefficients between health behaviors and polypharmacy after adjusting for multimorbidity. Even after this adjustment, health behaviors remained significantly related to polypharmacy. This suggests that, despite comorbidities acting as mediators, promoting healthy behaviors could potentially mitigate polypharmacy and its related adverse effects. Furthermore, due to the characteristics of the medical services in Korea, older patients with two or more conditions often utilize multiple healthcare facilities [37]. These results align with previous findings indicating higher healthcare utilization among females and those of advanced age with multiple comorbidities [19, 38].

The strengths of this study lie in its integration of representative data from the Korea National Health and Nutrition Examination Survey (KNHANES), alongside using nationwide data from the Health Insurance Review and Assessment Service (HIRA) and the National Health Insurance Service (NHIS). This represents the first instance of simultaneously analyzing health behavior information from survey data and medical service utilization information, thereby overcoming the limitations of previous studies that relied solely on claims data or surveys. By linking these two datasets, multidimensional variables such as health-related behaviors (e.g., alcohol consumption, smoking, regular walking, health screening) and socioeconomic information (e.g., economic status by income, private medical insurance enrollment) were added, enabling the analysis of significant factors related to polypharmacy and the resulting healthcare cost burden in older adults. Additionally, unlike previous studies that relied on self-reported medication reviews for key variables like polypharmacy [21], this study uses claims data, providing more reliable results regarding the relationship between health behaviors and polypharmacy.

Nevertheless, this study has several limitations. Firstly, our study relied on self-reported health behaviors obtained from the KNHANES, and recollection bias cannot be ruled out. However, many previous studies have used self-reported surveys to explore the relationship between health behaviors and healthcare utilization [21, 35]. Studies investigating the relationship between typical behavioral patterns and healthcare utilization have used traditional health behaviors such as physical activity, smoking, and Mediterranean Diet Adherence Screener as key variables [20, 21]. This study, however, was based on behavioral scores derived from the nationally representative KNHANES, which lacks dietary quality data. Secondly, the criteria for polypharmacy was defined based solely on prescription claims data of from older adult patients, potentially resulting in missing data from Korean herbal medicine and over-the-counter medications, which older adults in Korea rely on [39, 40]. Thirdly, this study focused solely on analyzing the association between polypharmacy among healthcare utilization services associated with protective health behaviors. However, for older adult patients, a significant portion of medical service utilization includes outpatient medication prescriptions, emergency department visits, and hospitalizations. Therefore, this limitation underscores the need for further research to address these comprehensive effects by investigating polypharmacy, medical expenses, and various forms of medical service utilization based on clustering or composite scores of protective health behaviors. Additionally, due to our study’s limited sample size, we could not proceed with medication-specific grouping. Further research is needed to focus on specific types or classes of medication, such as cardiovascular drugs.

Regardless, by analyzing health behaviors, socioeconomic factors, underlying medical conditions, and corresponding prescription rates, this study offers insight into the relationship between health-related behaviors and polypharmacy, a key healthcare service utilization in public health. Considering that polypharmacy has a more than 50% prevalence among older adults, the transition to multimorbidity must be prevented and managed through health behavior interventions among adults aged 65 to 79.

## Conclusions

This study corroborates that protective health behaviors such as obesity and smoking are risk factors for polypharmacy, and a higher composite score of protective health behaviors is associated with a reduced risk of polypharmacy. Prevention policies must take a comprehensive view, recognizing geriatric-centered management of health-related behaviors and well-being.

### Electronic supplementary material

Below is the link to the electronic supplementary material.

Supplementary Material 1

## Data Availability

The data that support the findings of this study are available from the Korea Healthcare Bigdata of the Ministry of Health and Welfare, which were used under license for the current study, and so are not publicly available.

## Abbreviations

ATC: Anatomical Therapeutic Chemical
BMI: Body Mass Index
CCI: Charlson Comorbidity Index
