# Supplementary figures and images for: An analysis of protective health behavior and polypharmacy among older patients: a nationwide cohort study
Source: BMC Geriatr. 2024 Jul 30;24:637. doi: 10.1186/s12877-024-05207-7 (PMC11290277; doi:10.1186/s12877-024-05207-7)

**
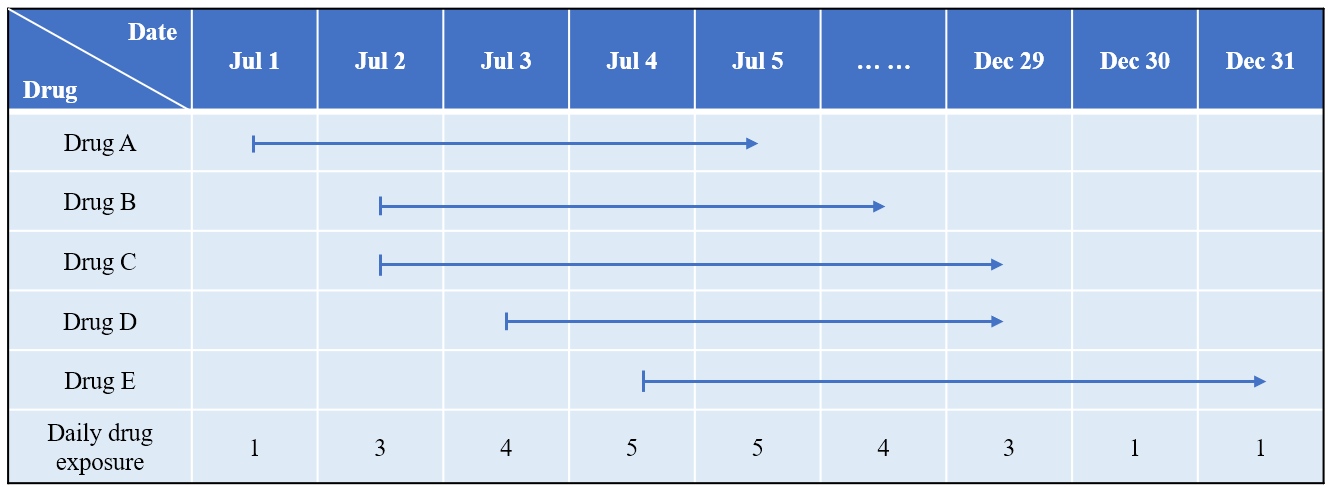
Appendix Figure 1. Definition of number of drugs and days in polypharmacy therapy**

Supplement: Supplementary file 1 — Supplementary Material 1 [file 12877_2024_5207_MOESM1_ESM.docx]
